# Conceptualising ‘Undue Influence’ in Decision-Making Support for People with Mental Disabilities

**DOI:** 10.1093/medlaw/fwaa041

**Published:** 2021-06-23

**Authors:** Jillian Craigie

**Affiliations:** Centre of Medical Law and Ethics, Dickson Poon School of Law, King’s College London, London, UK

**Keywords:** Disability, Discursive control, Mental capacity, Rights, Undue influence, Vulnerability

## Abstract

A crucial question in relation to support designed to enable the legal capacity of people with mental disabilities concerns when support constitutes undue influence. This article addresses this question in order to facilitate the development of law and policy in England and Wales, by providing a normative analysis of the different approaches to undue influence across decisions about property, contracts, health, finances, and accommodation. These are all potential contexts for supporting legal capacity, and, in doing so, the article compares approaches to undue influence that are rarely considered together.

Drawing on the UN Convention on the Rights of Persons with Disabilities, third sector and public body documents, and law in England and Wales, the analysis identifies six models that conceptualise undue influence in terms of: modes of influence; an overborne will; an inference from the situation; an overborne will understood as a mental incapacity; an overborne will in connection with vulnerability; and impaired discursive control. This final approach is a novel proposal for understanding undue influence. The analysis highlights key policy-relevant issues that distinguish the models, and generates a deliberative framework for navigating them, with the ‘overborne will’, ‘inference-based’, and ‘discursive control’ models identified as potentially fitting for the support context.

## I. INTRODUCTION

One of the significant shifts required by the UN Convention on the Rights of Persons with Disabilities (CRPD) is a move away from substituted decision-making based on mental impairment or mental incapacity, and towards the enabling of legal capacity through support.^1^ There remains controversy surrounding the interpretation of the Convention, regarding the degree of shift required away from substituted decisions.^2^ Nonetheless, the concept of supported decision-making now features prominently in policy documents regarding legal capacity and mental disability, with the CRPD regularly cited as grounding its use.^3^

Concerns about undue influence can arise in a wide range of decision-making situations, but they are a particular concern in the context of support designed to enable legal capacity for someone with a mental disability. Supporters may be required to be significantly involved in decisions,^4^ legal frameworks for supported decision-making may give supporters powers that could be misused,^5^ and these factors may combine with intrinsic factors related to the supported person’s disability to mean that they are at greater than normal risk of undue influence. As a result, while a shift from substituted to supported decisions provides an opportunity for empowerment—with the person moving from decisions being made on their behalf to being the legally recognised decision-maker—this empowerment may be curtailed by the inappropriate involvement of a supporter. When this is the case, the sense in which the supported person is the ‘decision-maker’ is, arguably, limited. Furthermore, if legal frameworks allow supported decisions to have adverse effects,^6^ then there is a risk of harm for the decision-maker in connection with undue influence, which may involve the supporter using the role to their own advantage. An undue influence remedy is therefore an important element within law for supported decision-making.

However, this area is still evolving and in many jurisdictions, including England and Wales, an approach to undue influence for the context of the support relationship is yet to take shape in law reform.^7^ Third sector and public body organisations have been active in making recommendations regarding support for legal capacity, and some of these have discussed undue influence or related issues. In the academic literature, discussions about the Convention and its implications for frameworks governing legal capacity, have begun to consider how undue influence might be understood in this space between substituted decisions and decisions made independently without legal regulation.^8^ The present article investigates this issue in depth, exploring how undue influence should be understood in law and policy for supported decision-making in England and Wales, against the normative background of the CRPD. However, the conceptual nature of the analysis is likely to facilitate its relevance in other jurisdictions.

The notion of undue influence is well established in England and Wales, but it has been understood in different ways in different parts of the law. The article uses these various approaches, as well as those outlined by the Committee on the Rights of Persons with Disabilities and third sector or public body organisations, to distinguish six models for conceptualising undue influence. An analysis of each model in relation to the normative perspectives associated with the Convention, generates a deliberative framework for policy-makers and provides recommendations concerning the fit of each model for the context of supported decision-making.

The article begins with a digression about what constitutes appropriate support in accordance with the CRPD, which outlines the normative tensions underlying questions of support and legal capacity. It then examines the approach to undue influence set out in General Comment No. 1 on the CRPD, which is used to identify the first, *modes of influence*, model. Documents from third sector and public body organisations are analysed to present a range of views on undue influence from an advocacy perspective and to introduce the idea of a *will-based* model, before the article then moves to law in England and Wales. Property law and contract law provide examples of a *will-based* approach—the overborne will standard—and are used to distinguish a third, *inference-based*, model. These are further explored in cases concerning medical consent, and here a fourth model is identified, adopting a *will-based mental incapacity* approach. The fifth, *will and vulnerability-based*, model is drawn from cases invoking the High Court’s inherent jurisdiction in the context of decisions about accommodation, cohabitation, and finances. Finally, the article considers the approach to conceptualising ‘undue pressure’ in the context of support to enable mental capacity under the Mental Capacity Act 2005 (MCA), and proposes a sixth model derived from a *discursive control* account of decision-making freedom.

While the meaning of ‘support’ for legal capacity is left open in the CRPD, it is understood to include support in the decision-making process, and this is the context in which the meaning of undue influence will be explored below.^9^ ‘Support’ for legal capacity is also used to refer to functions such as the enacting of decisions, but these other dimensions of support tend not to involve concerns about undue influence and so are not the focus here. For the purpose of this article, the terms ‘support in decision-making’, ‘decision-making support’ and ‘support’ refer to support in the decision-making process, for people with mental disabilities including mental ill health, where decisions involve legal standing or have legal effect. This definition is admittedly vague in relation to what support involves in practice—an issue that will be picked up in the following section. The article does not distinguish between contexts where support is provided by friends and family, and those where support is provided by advocates or legal, financial or clinical professionals.

## II. NORMATIVE UNCERTAINTY CONCERNING BOTH ‘APPROPRIATE SUPPORT’ AND ‘UNDUE INFLUENCE’

One way to approach the question of what constitutes undue influence in the decision-making support context would be to ask what ‘appropriate influence’ involves, and to understand influence as undue when it is not appropriate. These concepts are connected because they cannot overlap. An instance of support cannot be appropriate *and* constitute undue influence. However, there are at least two reasons for not using the idea of appropriate support as a starting point for developing an account of undue influence in this area.

The first is that undue influence need not begin where appropriate support ends. As Ryan Morgan and James Devenney have noted in relation to consent in a medical context, a significant gap exists between what is deemed appropriate practice according to guidance on consent procedures—where pressure of any kind from a professional, on the patient, is considered unacceptable—and the law concerning undue influence in this area, which allows for a considerable degree of pressure without a professional’s influence being deemed undue.^10^ Morgan and Devenney identify this as a problematic feature of law and guidance in this area. The present article merely notes that current regulation in at least one area of medical consent has adopted an approach whereby appropriate practice and undue influence do not meet. This means that the usefulness of an answer to the question of what constitutes appropriate support may be limited in drawing the boundaries of undue influence.

The second reason is that there is, arguably, as much uncertainty about what constitutes appropriate support, as there is about what constitutes undue influence. The analysis of policy documents below illustrates that a shared understanding of what constitutes good practice in support to enable the legal capacity of people with mental disabilities, is currently lacking. In these documents, we see different positions on how much, and what kinds of influence should be endorsed in decision-making support. One reason for uncertainty concerning what support for legal capacity should involve in practice, is tensions within the values that underpin the CRPD and therefore also the developments flowing from it.^11^ What seems like a good model of support will depend on a view about its purpose, which will reflect a set of underlying evaluative commitments in this area. In this way, normative uncertainty plays a role in the practical uncertainty concerning the meaning of both ‘appropriate support’ and ‘undue influence’. A sketch of the normative terrain is provided here to illustrate this background complexity, and to situate the analysis throughout the article.^12^

If legal recognition *per se* and non-interference are held to be the most valuable ends in this context, then enabling legal capacity through support should focus on eliciting the supported person’s preferences or decisions, and advocating on their behalf—giving the person’s expressed wishes legal effect, whatever they may be. An informed consent model of support might fit with this evaluative outlook, with its minimal interference in decision-making. On the other hand, the idea of getting involved in the reflective process, challenging the person or influencing their decision through support, seem likely to raise concerns from this evaluative position.

However, if the importance of legal capacity is understood, instead, to be grounded in the value of recognising decisions that reflect the person’s individuality or self, then models of support to enable legal capacity will look somewhat different. Viewed from this evaluative position, support will not always involve giving legal effect to the person’s currently expressed wishes. It may include challenging the person,^13^ working with the person to develop their identity and commitments,^14^ or enabling psychological capacities such as self-confidence or imagining future consequences, insofar as these are understood to facilitate self-reflective decisions. In one way or another, support may involve more interference than would be considered appropriate from the first evaluative stance.

While the Convention does not distinguish between these interpretations, its goals and guiding principles provide a starting point for navigating them. Article 1, outlining the Convention’s fundamental goals, begins with the ‘full and equal enjoyment of all human rights and fundamental freedoms’, respect for ‘inherent dignity’ and ‘full and effective participation in society’ for all. Article 3, which provides the Convention’s guiding principles, foregrounds ‘Respect for inherent dignity, individual autonomy including the freedom to make one’s own choices, and independence of persons’. While there is room for discussion about what actions would realise ‘respect for inherent dignity’, for example, the prominence given to the language of rights, freedoms, autonomy, choice and independence may be taken to indicate that the first of these evaluative outlooks more closely indicates what decision-making support should involve in accordance with the Convention.

Nonetheless, the emphasis in the Convention and General Comment No. 1 on the recognition of ‘will and preferences’, arguably allows room for the second evaluative perspective. Much depends on how the phrase ‘will and preferences’ is interpreted.^15^ This perspective might also draw support from discussions of other parts of the Convention including Article 16 on ‘Freedom from exploitation, violence and abuse’.^16^ The concern about the second evaluative position and its related ideas about support in practice, is that it could be used to justify constraining the full enjoyment of equal legal recognition for people with mental disabilities. It might compromise the paradigm shift envisaged by many commentators, away from seeing people with mental disabilities primarily as recipients of care,^17^ by allowing a continuation of the paternalism that has dominated past practice, merely described in another way.

However, these ideas about what good decision-making support might involve from the second evaluative perspective, describe interventions that would not seem out of place in most ordinary situations where one person is enjoined to support another’s decision. If we were to learn that someone had asked a family member or friend for support—for example, in a decision about contraception or taking out a personal loan—we would surely think that the supporter had done an inadequate job if all they did was provide the relevant information in an appropriate form, elicit the person’s current wishes, and ensured these were given effect. Drawing on a considerable philosophical literature, Camillia Kong argues for the importance of relationships and dialogue in promoting key competencies for autonomy.^18^ Likewise, Wayne Martin and colleagues suggest that the influence of others in decision-making ‘might be regarded as positive, beneficial and autonomy-enhancing, and therefore as a necessary feature of any supported decision-making regime’.^19^

This set of considerations presents a dilemma. Some will argue that only the first evaluative stance will properly protect people with mental disabilities from unwarranted interference in the exercise of their legal capacity—providing equal recognition before the law. The highest value must, therefore, be placed on non-interference, and support models must reflect this. However, adopting this approach seemingly fails to provide people with mental disabilities the kind of support we think that anyone deserves in the context of a significant decision.

More controversially, it does not allow for recognition that mental disabilities are often associated with impaired psychological functioning that goes beyond what most people face through much of their life; for example, in the case of memory impairments associated with dementia. A social model of disability would seem to allow, or even require, that support is provided to minimise the disability associated with psychological impairments. Not providing support that addresses the impairment—enabling functioning in one way or another—arguably imposes disability on the person, according to a social model such as that as endorsed by the CRPD.^20^

This way of thinking might be contested at least two ways. A question could be raised about whether the differences in functioning (whatever is identified as the psychological impairment, for example, a memory impairment) are as clear cut or significant as they are said to be. Eilinior Flynn and Anna Arstein-Kerslake seem to make an argument along these lines when they suggest that our understanding of human decision-making remains limited and that recent developments have questioned the role traditionally assigned to ‘logic’ in decision-making.^21^ The idea appears to be that the psychological impairments Flynn and Arstein-Kerslake have in mind do not relevantly impair functioning at the level of decision-making. If this is true, then support that aims to address the psychological impairment would not seem justified; support should instead focus on advocacy for the legal recognition of the person’s expressed wishes.

Another possible response would be to agree that what are identified as psychological impairments do have an impact on decision-making, but to argue that they should, nonetheless, be recognised as part of the person’s own way of living their life—their individuality. This way of thinking draws on a neurodiversity perspective, which has emerged in recent years as a way of reframing the idea of, for example, being autistic or bipolar.^22^ If this reconceptualisation in terms of identity rather than disorder is adopted, the appropriate response would seem to be recognition of the differences in psychological functioning and decision-making as part of the person, rather than support that aims to ameliorate those differences.^23^

The work of Mindy Chen-Wishart underscores the important role for values in law concerning undue influence. She shows, for example, how societal norms concerning the parent–child relationship shape what circumstances justify an inference to undue influence when an adult child guarantees a parent’s debt. A finding of undue influence is made when the standard expectations of the relationship are transgressed. In a society where family interests and deference to parents are highly valued, a decision that reflects these norms is unlikely to be deemed unduly influenced.^24^ According to Chen-Wishart, this is not merely a matter of what happens in practice, it is the transgression of these norms that *justifies* state interference to vitiate a transaction on grounds of undue influence.^25^

In line with this account, and noting the tensions outlined above, one crucial question for policy makers concerns the normative foundations upon which an approach to undue influence for the support context is to be developed. What values should be accepted as constituting the relationship between the supporter and supported person, and, crucially, what do they mean in practice? In the same way that family interests or deference to elders might be valued in certain social contexts, what features should be considered central to support relationships that aim to enable the legal capacity of people with mental disabilities? This is presented as an open question—against the background of the positions outlined above—which is returned to throughout the article.

## III. THE CRPD ON UNDUE INFLUENCE: INTRODUCING A ‘MODES OF INFLUENCE’ MODEL

The CRPD requires that measures ‘that relate to the exercise of legal capacity’, including support measures, ‘are free of conflict of interest and undue influence’, with the caveat that any protections must be commensurate to the degree of impact on the person’s rights and interests (Article 12(4)). On the face of it, this restriction on safeguards seems non-controversial. However, what it means in practice depends significantly on which rights and interests are given emphasis, and whether it is rights or interests that are given precedence. As a discussion paper from the Office of the Public Advocate in Victoria, Australia, noted:

‘Appropriate external monitoring or accountability requirements may alleviate the [possibility of undue influence and other risks] but thereby impinge on the freedom of action of the person with a disability. The balancing of freedom and protection is thus an issue in supported decision-making as in all other measures designed to promote the rights of people with disabilities in our community.’^26^

The Convention does not elaborate further on undue influence and, as argued above, its normative stance remains open to interpretation in relation to this balancing question. General Comment No. 1 from the Committee on the Rights of Persons with Disabilities acknowledges that a reliance on the support of others may increase the risk of undue influence beyond that present in any decision-making situation. However, the definition it adopts is notably narrow:

‘Undue influence is characterized where the quality of the interaction between the support person and the person being supported includes signs of fear, aggression, threat, deception or manipulation.’^27^

The caveat in the Convention—delimiting protections based on the rights and interests of the person being supported (Article 12(4))—is applied again in General Comment No. 1. However, in this context it is given a little more substance, with reference made only to the ‘right to take risks and make mistakes’.^28^

The narrow scope of the characterisation in General Comment No. 1 can be understood in terms of two features. One is its focus only on certain modes of influence: the generation of fear, aggression, threat, deception, manipulation. The other is that these ways of influencing tend to involve wrongdoing on the part of supporter,^29^ and a likelihood of harm to the supported person.^30^ This approach to conceptualising undue influence is the first model identified in this article. Its key features form central themes throughout this analysis. They are in tension with the paradigmatic approach to undue influence in England and Wales, as will be explored below. However, a ‘modes of influence’ model is reflected in the first of the policy documents to which the article now turns.

## IV. THIRD SECTOR AND PUBLIC BODY PERSPECTIVES ON UNDUE INFLUENCE: INTRODUCING A ‘WILL-BASED’ MODEL

An examination of third sector and public body recommendations and guidance on decision-making support in response to the CRPD found that few documents provided an account of how undue influence should be understood or approached in this context.^31^ Publications from three organisations, in Scotland and Australia, which address this or related issues are discussed in this section. They present a range of perspectives in connection with undue influence, including a ‘modes of influence’ approach, and what will be called a ‘will-based’ model.

Beginning with a perspective that is broadly in keeping with General Comment No. 1, the Mental Welfare Commission of Scotland has provided an indication of the approach to undue influence that it would advocate. As part of a proposal for a graded guardianship system, it calls for recognition that ‘*influence* may often be benign’.^32^ It recommends that the approach to undue influence should focus on upholding the principle of non-discrimination by looking to areas of law unrelated to disability,^33^ and a case study in the organisation’s ‘Supported Decision Making’ good practice guide^34^ provides insight into how undue influence might be understood and managed on such an approach—although in this example it is not a supporter but someone else who is suspected of undue influence.

In the case study, a young woman with a mild learning disability is befriended by a group who are into tattoos. At their urging, she decides to get a tattoo and is persuaded by a tattoo artist connected to the group to have several large tattoos done. The cost will compromise the woman’s ability to pay her bills, and there is a concern that she may have been subject to undue influence for financial gain, with the group telling her she could remain with them only if she has the tattoos done. The prescribed support remedy is that

‘steps are taken to ascertain whether or not she has been presented with all the options and the benefits and disadvantages of getting the tattoos, and a suitable place and amount of time to reflect on these, so that she was able to make a fully informed decision’.^35^

The primary concern in this case is about manipulation, which falls within the narrow characterisation of undue influence provided in General Comment No. 1. The focus is a mode of interaction, which involves wrongdoing on the part of the influencing party, and, in this case, a likely financial harm for the woman. The proposed support response is also in keeping with an interpretation of the Convention's normative commitments that prioritises non-inferference, with the guide recommending an informed consent model of support. This intervention is relatively minimal, with support not including interventions such as helping the woman manage interpersonal relationships or capacity building in areas such as self-confidence.

The second example of relevant policy is a piece of guidance for direct support workers produced by Scope in Victoria, Australia. It does not use the language of undue influence. However, the document highlights the importance of remaining ‘neutral’ in a way that seems to respond to this kind of concern:

‘Being neutral means not influencing the person to make one decision over another because of your own beliefs, or because it is the decision you would make if you were in their shoes.’

‘If you think you can’t avoid influencing someone, find someone else to provide support for that decision.**’**^36^

One way of understanding this guidance from Scope is to see it as ensuring the self-authorship of the person’s decision—a purpose that is arguably shared by law concerning undue influence. However, based on this guidance, an act of support might be deemed inappropriate and therefore potentially a case of undue influence, even if it is well-intended and does not involve threat, manipulation or signs of fear. Moreover, it may be considered inappropriate despite the resulting decision being good for the supported person, at least from a financial or healthcare perspective, or even from the supported person’s perspective in retrospect. The concern being addressed in this guidance is, therefore, not about the supported person being wronged in any of the ways listed in General Comment No. 1, or harmed by the outcome of a decision, and this points to a wider set of potential concerns in this area.

Applying this thinking from Scope, it could be argued that the influence of a supporter might be undue simply because they are too influential. There is too much of their perspective reflected in the person’s decision. This way of thinking about undue influence, arguably, also connects with a position in General Comment No. 1, that supports to enable legal capacity must give expression to the supported person’s will and preference.^37^ This raises a question about whether the approach to undue influence in the context of decision-making support flowing from the Convention, should be widened beyond the characterisation provided in General Comment No. 1, to include concerns of this kind.

The third policy document outlines a supported decision-making framework from the advocacy organisation ‘People First’ in Scotland.^38^ It diverges from the previous two approaches in terms of the degree of influence that is considered acceptable in the practice of support. It also outlines an account of undue influence in the context of supported decisions, that appears to be wider than that provided in General Comment No. 1.

On the question of what constitutes appropriate support, the People First framework appears to take a broader approach than both Scope and the Mental Welfare Commission for Scotland, by proposing that ‘Good support will challenge the individual but never take away their right to make decisions that reflect their will and preference’.^39^ This vision of support goes beyond an informed consent model in which the supporter’s role is to help the person understand possible consequences and ensure the person has sufficient time to reflect, and it expresses no concerns about the idea that the supporter may influence a decision. Rather, it endorses the idea that support should involve challenging the person being supported.

In relation to undue influence, the People First framework also adopts a wide definition, proposing that influence is undue when a person is made ‘to act in a way that is different to their free will’.^40^ The framework gives aggression, threat, manipulation, withholding or falsifying information and giving unsound advice, as potential modes of undue influence, especially where there is a conflict of interest. However, this list is not presented as exhaustive, and this definition appears to leave open the possibility that support might constitute undue influence despite being well-intended and not involving a conflict of interest or a risk of harm to the person being supported. The supporter might simply have too much influence, such that the resulting decision cannot be said to reflect the supported person’s free will. This approach to undue influence, focused on the impact of influence on the freedom of the person’s will, provides an example of the second approach to understanding undue influence identified in this article—a ‘will-based’ model.

These documents crystallise two issues relevant for the development of law and policy for supported decision-making. The first concerns whether undue influence in the context of support is more appropriately characterised using a modes of influence model or in terms of the consequences of support in a will-based model. The second is about whether undue influence in this context must involve a wrongful action, or whether it may be ‘innocent’.^41^ The following sections explore these issues within the jurisdiction of England and Wales, beginning with the will-based approach found in property and contract law.

## V. UNDUE INFLUENCE IN PROPERTY LAW AND CONTRACT LAW: ELABORATING ON A ‘WILL-BASED’ MODEL

The most fully developed approach to undue influence in England and Wales is found in law concerning property and contracts. As set out in the leading case of *Etridge*, undue influence is a doctrine that was developed to ensure that ‘the influence of one person over another is not abused’.^42^ Lord Nicholls goes on to explain that the doctrine recognises that people legitimately seek to influence others as part of everyday life; nonetheless, the law ‘has set limits to the means properly employable for this purpose’,^43^ and when a consent is secured by influence that is deemed undue, the transaction is set aside.

Providing a definitive account of what makes influence undue is said to be impossible because of the wide-ranging circumstances in which transactions occur. However, the essence of the doctrine holds that influence is undue:

‘whenever the consent thus procured ought not fairly to be treated as the expression of a person's free will.’^44^

This area of law may be one source for the reference to ‘freely willed’ decisions in the People First supported decision-making framework.^45^ The People First document does not explore this idea further. However, in English and Welsh property and contract law, questions of whether a consent is an expression of the person’s free will are assessed by asking whether their will was ‘overborne’. The classic statement of this approach was set out by Sir JP Wilde in *Hall v Hall*, a case concerning the making of a will:

‘Persuasion, appeals to the affections or ties of kindred, to a sentiment of gratitude for past services, or pity for future destitution, or the like, these are all legitimate, and may be fairly pressed on a testator. On the other hand, pressure of whatever character, whether acting on the fears or the hopes, if so exerted as to overpower the volition without convincing the judgment, is a species of restraint under which no valid will can be made. … these, if carried to a degree in which the free play of the testator's judgment, discretion or wishes, is overborne, will constitute undue influence, though no force is either used or threatened. In a word, the testator may be led but not driven; and his will must be the offspring of his own volition and not the record of someone else's.’ ^46^ ’

Understood in this way, influence is undue if the will of the person was overborne, and this is the case when the influence of another, ‘overpower[s] the volition without convincing the judgment’; such that the decision is not the product of the person’s own volition, but ‘the record of someone else's’.

On this approach, threats do not automatically constitute undue influence, and seemingly mild forms of persuasion might. As Lewison J. explained in *Edwards (Deceased)*, the mental strength of the person making the decision is crucial in assessing the impact of a particular interaction:

‘The will of a weak and ill person may be more easily overborne than that of a hale and hearty one. As was said in one case simply to talk to a weak and feeble testator may so fatigue the brain that a sick person may be induced for quietness' sake to do anything.’^47^

The modes of influence that could be undue are therefore very wide—even to talk to someone in a weak mental state can amount to undue influence. In this way, the decision-maker’s physical and mental state may be relevant to the assessment of the relationship between their expressed wishes and their will.

Based on these observations, several points can be made regarding whether a ‘modes of influence’ or ‘will-based’ model is more appropriate for the context of decision-making support. A focus on modes of influence has the advantage of providing clear guidance for practice.^48^ However, concerns about inappropriate influence in this context will not be limited to certain kinds of influence. The most challenging cases in this area are likely to be situations that do not involve obviously problematic modes of influence. Providing a structure that addresses these cases would seem to require a more nuanced approach along the lines of a will-based model. This approach also has the apparent advantage of fitting well, at least *prima facie*, with the emphasis on the expression of ‘will and preference’ in Article 12 of the CRPD.^49^

However, two concerns might be raised about applying the overborne will standard to the context of decision-making support. One is whether allowing the decision-maker’s mental and physical state to play a role in determining whether an influence is undue, runs the risk of unjustified discrimination against people with mental disabilities. Might the person’s disability, in effect, form the basis of undue influence findings in place of a proper assessment of whether their will was overborne? Although disability is neither directly or indirectly necessary for undue influence within this approach, which allows it to be described as disability-neutral, this question regarding the law in practice seems important to consider.

Second is a concern about the degree of pressure that could be used by a supporter before their influence would be deemed undue. In accordance with the overborne will standard, influence would not be undue if the decision-maker’s judgement is brought around, and this would allow supporters a great deal of latitude in the influence they could have before a transaction or consent could be vitiated. The concern is that this approach might not provide sufficient protection against inappropriate interference by a supporter. This degree of influence seems unlikely to be endorsed by Scope, for example, given their guidance for support workers against influencing decisions. However, a recent Court of Protection case in England and Wales indicates a willingness in this context, to view a person’s expressed wishes as reflecting their own will, despite recognising that those wishes were adopted due to the very significant influence of another.^50^ The case involving a 22-year-old man with physical and intellectual disabilities, found that although the influence of his mother was pervasive, his expressed wishes against a residential rehabilitation stay were his own, and these were therefore accorded the relevant legal standing.^51^

This account of the meaning given to undue influence in English and Welsh property and contact law develops the analysis of a will-based model. It was argued that, for the decision-making support context, a will-based approach has important advantages over a modes of influence approach. Crucially, in avoiding a focus on modes of influence it allows a broader and more nuanced analysis that seems appropriate for the complexities of the support context. However, the idea of straightforwardly applying the overborne will standard brings to light two further policy-relevant issues. One concerns the risk of unjustified discrimination based on mental disability due to the weight potentially given to the decision-maker’s mental or physical state. The other concerns the degree of supporter influence that should be allowed before a decision can be vitiated. Considered against the normative background of the CRPD, a straightforward application of the overborne will standard might, arguably, allow an inappropriate degree of influence by supporters.

Within property and contract law, an alternative evidential route to a finding of undue influence is provided, which recognises the need to protect people in relationships where ‘the influence one person has over another provides scope for misuse without any specific overt acts of persuasion’.^52^ It allows undue influence to be inferred from the situation, and this is the third model identified for consideration in the support context.

## VI. UNDUE INFLUENCE IN PROPERTY LAW AND CONTRACT LAW: INTRODUCING AN ‘INFERENCE-BASED’ MODEL

An inference to undue influence, or ‘presumed’ undue influence, is established in property and contract law via a rebuttable presumption that arises (i) in relationships that involve a ‘presumption of influence’ where one person has a degree of influence or ascendency, and (ii) where the transaction ‘calls for an explanation’ because it cannot easily be explained by ordinary motivations such as friendship, relationship or charity.^53^ Once the presumption is established, the party who wants the transaction to stand must show that the position of influence was not abused—typically by showing that the decision-maker had independent legal advice—or the transaction will be set aside.

Perhaps the most compelling reason for thinking an inference-based model may be appropriate for the decision-making support context, is that the support relationship has the features that are used to establish a presumption of influence in existing law. This element is automatically met by certain relationships, including parent/child, spiritual advisor/devotee and doctor/patient relationships, on the basis that they involve ‘trust and confidence, reliance, dependence or vulnerability … [or] … ascendency, domination or control’.^54^ The relationship between a person with a mental disability and the person supporting their legal capacity can also be assumed to share these features. Trust and confidence seem essential on the part of the supportee; this kind of support relationship will, by definition, involve a degree of dependency, and mental disability is, arguably, associated with vulnerability.^55^ Like the above relationships, the support relationship ‘may be such that, without more, one [person] is disposed to agree a course of action proposed by the other’.^56^

If an inference-based approach was adopted in the decision-making support context, and if the ‘presumption of influence’ element was automatically met by the support relationship,^57^ the crucial issue determining whether the influence of a supporter was undue would concern whether the decision calls for an explanation. In relation to this second element, a key area of controversy concerns the relevance of the transaction outcome; for example, whether it must be ‘improvident’ or ‘substantively unfair’ to call for an explanation.^58^  *Etridge* established that although undue influence need not involve a disadvantageous outcome for the influenced person,^59^ presumed undue influence is likely to involve a serious disadvantage because such circumstances will require a compelling explanation.^60^ However, commentators have highlighted that this remains an area of uncertainty in practice, with some cases post-*Etridge* indicating that an unfair or harmful outcome is necessary to infer undue influence.^61^ Analysing the case law, Mindy Chen-Wishart urges that, ‘contrary to the predominant view, substantive unfairness is of the essence of undue influence’.^62^

Interwoven with discussions about the relevance of the transaction outcome—and sometimes not distinguished—is a related issue about whether wrongdoing by the influencing party is necessary for establishing that a transaction calls for explanation. Academic debate on this issue has taken the form of a disagreement about whether undue influence should be conceptualised in terms of impaired consent (a ‘claimant-sided’ view) or wrongful conduct (a ‘defendant-sided’ view). Peter Birks and Chin Nyuk Yin have argued for a claimant-sided view, based on the importance of allowing that undue influence might be ‘innocent’.^63^ This position has been criticised on grounds that an impaired consent view involves a ‘fiction that the claimant gave no effective consent despite acting knowingly and intentionally’; as well as a ‘somewhat insulting characterisation’ of the influenced person’s decision-making as impaired.^64^ While rejecting the dichotomy within this jurisprudential debate, Chen-Wishart observes that although ‘there is no requirement of bad conduct or bad faith to infer undue influence in English law’, words indicating wrongdoing are frequently seen in judgments.^65^

The relevance of outcome and wrongdoing must be considered separately in the development of an approach to undue influence in decision-making support, because they will sometimes come apart. In relation to property and contracts, the two issues are often connected because an unfair or disadvantageous outcome will indicate wrongdoing by the influencing party when they stand to benefit. However, in less conventional undue influence cases, the issues of outcome and wrongdoing arise independently. In the medical consent case of *Re T*, discussed below, a mother’s influence on her daughter’s refusal of a blood transfusion was deemed undue.^66^ The likely outcome of death was undoubtedly disadvantageous, but it is hard to see that the mother’s pressure involved bad conduct given that it accorded with her religious beliefs, which were presumably pressed in good faith.^67^

Similar situations, involving a disadvantageous outcome but no bad conduct or bad faith on the part of influencing person, seem likely to arise in the decision-making support context. A family member acting as a supporter to someone with an intellectual disability might encourage them to accept an arranged marriage, to put an end to a romantic relationship with someone who does not have a mental disability,^68^ or to consent to sterilisation. A partner acting as a supporter might encourage the person not to use contraceptives. In some cases of these kinds, the decision might foreseeably result in a significant harm for the supported person, while involving only good intentions on the part of the supporter. In such contexts, the influence might nonetheless be deemed undue, as was the case in *Re T*.

Moreover, it might be argued that in the decision-making support context, a finding of undue influence should be possible when the situation involves neither supporter wrongdoing nor a disadvantageous outcome for the supported person. Concerns about the influence of a supporter seem likely to often take this form, if it is assumed that supporters will largely be well-meaning people wanting the best for the person they are supporting. The basis for the concern in such cases will be improper paternalism, with the supporter being accused of overstepping the remit of their role; for example, by pressing the person to make a financially prudent or health-promoting decision against their will. A question is therefore raised about whether these are situations that should be included within the protections provided by the undue influence remedy.^69^

This analysis suggests that an inference-based model may be appropriate for the context of decision-making support, given that a ‘presumption of influence’ applies almost by definition to the support relationship. If this model was applied, questions of inferred undue influence would turn on whether the supported decision calls for explanation. The answer typically concerns whether the decision results in disadvantage or unfairness for the influenced person, or whether it involved a wrongful act on the part of the influencing party, which raises a question about the suitability of these elements for the support context.

Two main considerations seem relevant regarding these features as elements of an inference-based model in the context of support. First, their inclusion would place a significant limit on the scope of protections against supporter influence. So long as the influence was well-intended, fair and in the decision-maker’s interests, it would not be undue. One risk, therefore, is that support may take the form of pressuring the person to make a decision that is in their best interests. The second consideration concerns the supported person’s ability to make risky or outright harmful decisions. If a supported decision risked significant harm, independent advice would be required or the decision could be set aside. The question for policy makers is whether such protection is ‘commensurate to the degree of impact on the person’s rights and interests’.^70^ What unifies these two concerns—one about paternalistic supporter influence and one about the right to make risky decisions—is their tension with the normative re-orientation envisaged in the CRPD, away from paternalistic responses to disabled people. However, if these two elements are to be avoided in the support context, an alternative account of when a supported decision ‘calls for an explanation’ would be required for an inference-based approach.

In England and Wales, the concept of undue influence has been applied beyond property and contract law in cases concerning, for example, decisions about medical treatment, accommodation and cohabitation. The following section analyses significant cases concerning consent in a medical context where a concern about inappropriate influence has been addressed. These elaborate further on the overborne will standard, and are used to identify a fourth model of undue influence to be considered in the development of an approach for the support context.

## VII. UNDUE INFLUENCE IN MEDICAL CONTEXTS: INTRODUCING A ‘WILL-BASED MENTAL INCAPACITY’ MODEL

In medical contexts, courts have held that the approach to undue influence in property and contract law does not straightforwardly apply. As Morgan and Devenney put it, ‘The doctrine of undue influence is “context specific”’.^71^ Slaughton L. J.’s justification for this position in *Re T*, was based primarily on the idea that a person guaranteeing a debt or making a will is not in the same position as a person making a decision about their own medical care.^72^ In *Mrs U*, a case concerning consent to the posthumous use of sperm in an IVF process, Lady Hale further distinguished this medical context, holding that,

‘none of the case law on undue influence in other contexts is particularly helpful. This is not like deciding upon the validity or enforcement of a will, gift or other transaction, which may have been procured by the undue influence of the person who will benefit from it. … Nor … is it like deciding upon the lawfulness of medical treatment. There are other justifications for performing life-saving medical treatment apart from the possession of an effective consent. There is no other justification for continuing to store human sperm.’^73^

These observations raise a question about the appropriateness of adopting a single approach to undue influence by a supporter, across the many kinds of decision that may be supported. Indeed, there is some divergence in approach within property and contract law, where undue influence in the context of making a will must be proven—it cannot be inferred.^74^ However, despite the above positions set out in *Re T* and *Mrs U*, commentators have noted that the approach applied in these two leading medical cases closely resembles the overborne will standard from property and contract law.^75^

*Re T* considered issues of both mental capacity and undue influence in the context of the High Court’s inherent jurisdiction, which at the time provided the basis on which an adult could be found to lack capacity.^76^  *Re T* concerned the refusal of blood products by a pregnant woman, T, who subsequently gave birth to a stillborn child, after which her condition worsened and she became unconscious. One of the issues considered was the influence of her mother, who belonged to the Jehovah’s Witness faith, which T had renounced. The initial court hearing found that although T was under the influence of her mother, this was not ‘of the kind which sapped her will and destroyed her volition’ and so was not undue,^77^ reflecting the framing of this issue from property and contract law. On appeal, it was found that there was ample evidence for a finding of undue influence.^78^ As lead judge, Lord Donaldson set out this position with reference to T’s weakened physical and mental state, her mother’s deep commitment to Jehovah’s Witness beliefs, and because the issue of blood transfusions was raised by T ‘out of the blue’ immediately after time alone with her mother.^79^

In providing guidance for healthcare professionals and bodies on how to respond to such situations, Lord Donaldson set out the following approach:

‘It is wholly acceptable that the patient should have been persuaded by others of the merits of such a decision and have decided accordingly. It matters not how strong the persuasion was, so long as it did not overbear the independence of the patient's decision. The real question in each such case is ‘Does the patient really mean what he says or is he merely saying it for a quiet life, to satisfy someone else or because the advice and persuasion to which he has been subjected is such that he can no longer think and decide for himself?’ In other words ‘Is it a decision expressed in form only, not in reality?’^80^

Lord Donaldson’s guidance goes on to emphasise the relevance, first, of the strength of the person’s will, including factors such as exhaustion, pain or depression; second, the relationship between people in question, with influence often being stronger in relationships such as that between a parent and child; and third, that certain kinds of arguments can be particularly forceful, for example, religious arguments. The importance of all three factors can be traced back to the property and contract case law.^81^

This guidance formed the basis for the approach applied in *Mrs U*.^82^ In forms completed prior to an IVF procedure, Mr U had provided consent that authorised Mrs U’s use of her husband’s frozen sperm to conceive, should he die. However, at the request of the IVF clinic—which had ethical concerns about the posthumous use of gametes—his consent was later withdrawn. Mr U died suddenly not long after, and Mrs U accused the clinic of securing the withdrawal of consent through undue influence.

The central issue was presented by the Court as whether Mr U’s will was ‘overborne so that the act of altering the form and initialling the alterations was done *in circumstances in which Mr U no longer thought and decided for himself*’.^83^ The finding that Mr U had not been subject to undue influence was appealed on the basis that the test used to establish undue influence was too strict.^84^ However, this argument was rejected, based largely on the importance of consent in law governing IVF procedures.^85^ All parties accepted that this was a situation in which undue influence had to be proven—it could not be inferred.^86^

The reasoning in *Mrs U* points to the importance of the situation and its normative features for determining how strictly the test for undue influence is applied. The Court of Appeal emphasised the importance of consent in IVF procedures as a justification for being ‘slow’ to make a finding of undue influence in this context.^87^ Reference was made also to the absence of ‘life-saving medical treatment’ as a factor to be considered in this context.^88^ Consistent with this indicated flexibility, a case concerning undue influence in decisions about cohabitation and accommodation identified ‘the preservation of human life’ as a factor that should be given significant weight.^89^

In relation to developing an approach to undue influence by a supporter, these cases point to a key issue concerning whether one conceptualisation is appropriate for the wide range of decisions that may be supported: financial, medical, and personal, among others. *Re T* and *Mrs U* provide some indication that a single approach might be applied to a range of decisions. Nonetheless, the Court’s reasoning in *Mrs U* suggests that certain features, such as whether a decision is life-ending, may justify applying the standard more, or less, strictly. If this kind of flexibility was adopted in relation to undue influence in decision-making support, further questions would concern how much flexibility is appropriate, and what kinds of consequences are relevant.

An importantly different approach to undue influence is seen in the medical case *Re A.*^90^ With the Mental Capacity Act 2005 (MCA) now in force,^91^  *Re A* signalled the possibility of linking questions of undue influence to the concept of mental incapacity. *Mrs A* concerned a woman with a learning disability and her decision about using contraception. The Court identified the dynamic between Mrs A and her husband, Mr A, as a central issue due to a concern that Mr A may have placed Mrs A ‘under so much pressure to refuse contraception that her capacity is impaired and/or that she is unable to exercise her free will in taking a decision for herself’.^92^

Within the MCA framework, a person lacks mental capacity when an ‘impairment of, or a disturbance in the functioning of, the mind or brain’^93^ renders them unable to understand, retain, use or weigh relevant information, or to express a decision.^94^ In *Re A*, the Court linked the concern about influence to the MCA by presenting the central issue as a question of ‘whether the influence of Mr A over Mrs A has been so overpowering as to leave her unable to *weigh up* the information and take a decision of her free will’.^95^ The Court concluded that due to ‘the completely unequal dynamic’^96^ within the couple’s relationship, Mrs’s A’s decision to stop taking contraception ‘is not the product of her own free will … she is unable to weigh up the pros and cons of contraception because of the coercive pressure under which she has been placed’, and so she lacked the mental capacity to make the decision.^97^ Several factors were identified as contributing to this situation, related to both Mrs and Mr A’s personalities and learning disabilities.^98^

Although a parallel was drawn with the framing of undue influence in *Re T*,^99^ in *Re T* the issues of mental capacity and undue influence were treated as independent. The approach in *Re A*, therefore, constitutes a significant shift, with the idea of an overborne will being understood in terms of a mental incapacity due to an inability to weigh relevant information. This represents a further, distinctive approach to undue influence—a ‘will-based mental incapacity’ model.

A number of considerations are raised by the possibility of applying this model to questions of undue influence in the support context. In favour of this approach, it might be argued that a mental incapacity-based model provides new ways of understanding undue influence at the psychological level, because it enables an exploration of what fine-grained capacities are relevant.^100^ However, understanding undue influence in terms of mental incapacity sits uneasily with the human rights perspective that is driving the shift to decision-making support. The tension is illustrated by the Committee on the Rights of Persons with Disabilities’ rejection of mental incapacity as a legitimate basis for denying legal capacity.^101^ Others have observed that the MCA’s ‘diagnostic threshold’, which makes an impairment or disturbance in the functioning of mind or brain an element of mental incapacity, is not disability-neutral.^102^ More fundamentally—beyond what the legal test requires—the notion of ‘mental incapacity’ is intuitively linked to impaired psychological functioning, and, as such, understanding undue influence as a mental incapacity locates the problem in the psychology of the influenced person. This explains why it may seem problematic to find that someone who is unduly influenced by an abusive partner lacked mental capacity as a result.^103^ Likewise, in a case of an adult child guaranteeing a parent’s debt. Together these concerns cast doubt on the fit of this model for the context of decision-making support.

The following section shifts to an examination of cases concerning other kinds of decision, primarily about accommodation and cohabitation, where the issue of influence has been raised. These are used to further explore a mental incapacity-based approach, and to illustrate the fifth model identified in this article.

## VIII. UNDUE INFLUENCE IN OTHER DECISIONS: INTRODUCING A ‘WILL AND VULNERABILITY-BASED’ MODEL

Lucy Series has observed that there is a ‘fundamental ambivalence’ in existing law on the issue of whether, and to what extent, the influence of others can impact upon mental capacity,^104^ and a number of recent cases support this claim. One example is *London Borough of Brent and NB*, which concerned a young man’s decision about a residential rehabilitation placement, which his mother was strongly against.^105^ In that case, the concerns about influence related to an ‘enmeshed’ relationship between *MB*, a young man with cerebral palsy, and his mother, NB,^106^ with their relationship described as ‘best summarised by his mother’s comment “we are one opinion”’.^107^ A clinical expert gave evidence that their relationship ‘impairs [MB’s] ability to conceive of separateness as viable and therefore impairs his ability to weigh the decision in the balance’.^108^ The solicitor representing another family member expressed uncertainty about whether this way of thinking about mental capacity was ‘capable of withstanding an analysis of capacity under MCA’.^109^ Despite concluding that due to the enmeshed relationship ‘MB is unable or unwilling to express views that differ from his mother’s’,^110^ the Court of Protection avoided the issue of influence and mental capacity by concluding that MB lacked the mental capacity to make the decision due to his brain injury.

More commonly, concerns about undue influence arising outside property and contract law since the MCA came into force, have invoked what remains of the High Court’s inherent jurisdiction, and this provides the fifth model identified in this article. Prior to the MCA, the inherent jurisdiction covered a wider set of issues than mental capacity—including undue influence, as seen in *Re T*—and it remains relevant where concerns have been raised about a person’s vulnerability, often despite them having mental capacity within the scope of the MCA. The courts have resisted defining this ‘vulnerable’ group.^111^ However, significant cases in this ‘jurisdictional hinterland’^112^ have involved people with ‘borderline’ mental capacity, who have been subject to undue influence, where these circumstances are said to remove the person’s ‘autonomy to make an important decision’.^113^ The cases of *Re L*^114^ and *Southend-on-Sea Borough Council and Mr Meyers,^115^* are used here to illustrate the approach found in this area of law.

*Re L* concerned an elderly couple’s relationship and living arrangement with their son,^116^ with the son’s influence over his parents providing the basis for the finding that they lacked capacity in these areas, under the inherent jurisdiction.^117^ The concerns about influence were raised due to reports of the son’s threatening and controlling behaviour.^118^ Outlining relevant law, the Court of Appeal described the circumstances that can invoke the inherent jurisdiction as including situations where a ‘vulnerable adult’s capacity or will to decide has been sapped and overborne by the improper influence of another’.^119^ Citing *Re SA*,^120^ and developing the approach set out in *Re T^121^* as well as property and contract cases, it was noted that influence may be subtle yet pervasive and powerful, especially where the mode of persuasion calls on ‘personal affection or duty, religious beliefs, powerful social or cultural conventions, or asserted social, familial or domestic obligations’.^122^ In such contexts, it was said that ‘very little pressure’ may be sufficient to overbear the person’s will.^123^

The case of *Mr Meyers* also concerned an elderly parent’s decision to cohabit with a son. Due to the son’s influence, it was deemed that Mr Meyers lacked the capacity to make this decision, invoking the High Court’s inherent jurisdiction, despite him having the mental capacity do so under the MCA.^124^ The court noted the absence of evidence of the son ‘forcing his father, either physically or verbally to act against his will’, but concluded that ‘the intensity of this relationship occludes Mr Meyers’ ability to take rational and informed decisions’.^125^ The son’s influence was said to be ‘insidious and pervasive’, triggering his father’s pronounced feelings of ‘duty, guilt, love and responsibility’ in a way that had become ‘confused and distorted’ due to their ‘enmeshed’ relationship.^126^ It was noted also that the influence exerted by the son was ‘malign in its effect if not its intention’,^127^ indicating that the harmfulness of the influenced decision played a role in the finding, but that wrongful conduct was not a necessary element of undue influence in this context.

In many respects the approach to concerns about influence in these two cases closely reflects the will-based approach from property and contact law, further developed in *Re T*. Unlike the will-based mental incapacity model seen in *Mrs A*, in *Re L* and *Mr Meyers* the construct of mental capacity was not used to analyse the question of influence. However, what distinguishes the approach in these two cases from that in *Re T* is the concept of vulnerability, which now plays a key role in invoking the inherent jurisdiction.^128^ This extra dimension warrants identifying this as a fifth approach to undue influence—a ‘will and vulnerability-based’ model.

Series has suggested that the will and vulnerability-based approach to undue influence found in inherent jurisdiction cases, may be appropriate for the context of support paradigms.^129^ However, Terry Carney raises the concern that the concept of vulnerability is ‘very woolly’—though Carney suggests this might be addressed through conceptual clarification.^130^ Relatedly, although the inherent jurisdiction has been utilised where the influenced person is not identified as having a disability,^131^ a question is raised about whether making vulnerability central to undue influence in the support relationship, runs the risk that the law will be implemented in a way that unjustly discriminates against people with mental disabilities. Michael Dunn and colleagues have argued that in leading inherent jurisdiction cases, ‘vulnerability’ is often constructed in terms of an inherent vulnerability, such as mental ill-health or disability, which is concerning because:

‘The vast majority of adults who fulfil the criteria for an inherent vulnerability [due to mental ill health or disability] … should not be judged to be *automatically* at heightened risk of being … unduly influenced, relative to other adults’.^132^

However, perhaps most crucially, it is unclear what the notion of vulnerability adds to the analysis of undue influence. The overborne will standard allows the influenced person’s physical and mental state, as well as the nature of the relationship, to be taken into consideration. These seem to be the features that underpin the identified vulnerability in the relevant inherent juristidction cases, and greater transparency would be achieved by making these the focus of questions concerning undue influence in the support relationship.

Together these observations suggest that a ‘will and vulnerability-based’ model carries risks when considered in relation to the emphasis on non-discrimination in the CRPD, without offering obvious advantages over the standard overborne will model.

## IX. ‘UNDUE PRESSURE’ IN SUPPORT TO DECIDE UNDER THE MCA, AND A ‘DISCURSIVE CONTROL’ MODEL

This final section examines an area of English and Welsh law that already provides for a form of decision-making support to enable legal capacity. Under the MCA, a person cannot be found unable to decide for themselves ‘unless all practicable steps to help him to do so have been taken without success’.^133^ This requirement aims to enable legal capacity within the current legal framework and, as such, its focus is on supporting the person to meet the MCA’s functional test—abilities to understand, retain, use and weigh information and express a decision.^134^ The implementation of support in this way appears to be in tension with the position adopted by the Committee on the Rights of Persons with Disabilities, against the use of mental incapacity as a basis for limiting legal capacity.^135^ Nonetheless, this context provides another account of undue influence to be considered in the development of policy and practice for decision-making support.

The MCA Code of Practice states that those providing this support must not ‘use excessive persuasion or “undue pressure”’.^136^  *Re T* and *Etridge* are given as authorities, which points to the relevance of the overborne will standard set out in these cases. However, the Code gives its own description of what could be deemed inappropriate influence in this context:

‘This might include behaving in a manner which is overbearing or dominating, or seeking to influence the person’s decision, and could push a person into making a decision they might not otherwise have made.’^137^

While the idea of an overbearing influence reflects the language used in *Re T* and *Etridge*, ‘seeking to influence’ a decision does not provide grounds for undue influence in accordance with the approach set out in these cases. The Code’s characterisation, therefore, indicates that a wider understanding of ‘undue’ may be appropriate in this context. This position resonates with that adopted by Scope, on the importance of supporters remaining neutral, and implies that a straightforward application of the approach in *Re T* or *Etridge* would allow supporters an inappropriate degree of involvement in decision-making.

However, to put this wider characterisation of undue influence into practice the idea must be given more substance. What does undue influence mean in this wider sense? The idea that any ‘attempt to influence’ would constitute undue influence is problematic because it could describe any act of support. The answer provided here is developed using Philip Pettit’s discursive control account of decision-making freedom.^138^

Drawing on Pettit's account, adapted for this context in my previous work, support would constitute undue influence when it limits either:

‘the [supported] person’s ability to respond to the pre-existing features of the options’, that is, considerations independent of the support process; or

‘the discourse that can be had between the supporter and the person being supported’ about those features.^139^

Understood in this way, support may legitimately involve, among other things, pointing out the possible consequences of the options and their likelihood; discussing those features in relation to what makes life meaningful or valuable to the person; and facilitating the person’s decision-making abilities, for example, using tools, training or therapy, insofar as these do not compromise the discursive relationship. However, support would involve undue influence when it introduces new, competing, considerations such as threats, incentives or emotional leverage. In addition, other attempts to ‘push the person’ into a decision may provide grounds for undue influence, along with aggression or the generation of fear, as these are likely to shut down discourse within the support relationship, and may also compromise the person’s internal discourse about the decision. Even the supporter expressing views such as ‘If I were you … ’ or ‘My advice would be … ’, if not invited, might constrain discourse about the pre-existing features of the decision and/or the discursive relationship between supporter and person who is being supported.

This account might be used to flesh out what constitutes an ‘overbearing’ influence in a wider sense that is appropriate for the support context, as indicated in the MCA Code. However, it is presented here as an alternative to a will-based standard, on the grounds that this curtailment of decision-making freedom may fall short of ‘overbearing’ the person’s will in any intuitive sense, and due to its focus on discourse rather than will. It provides an explanation of how the problematic interventions identified in the MCA Code, General Comment No. 1 and policy documents, limit decision-making freedom, without implicating supporter wrongdoing, a disadvantageous outcome, the supported person’s mental incapacity or their vulnerability. Rather, influence is deemed undue because the interaction restricts discourse about the decision, either within the supported person’s own thinking or their thinking with others. A discursive control account is, therefore, presented here as a sixth, and novel, model of undue influence.

One question raised is whether the discursive control model would too readily allow supported decisions to be vitiated. Here the normative issues highlighted at the beginning of the article, have a critical role to play.

## X. A DELIBERATIVE FRAMEWORK FOR DEVELOPING AN APPRAOCH TO UNDUE INFLUENCE FOR THE SUPPORT CONTEXT

The above analysis generates a framework for reflecting on how undue influence should be conceptualised in relation to those responsible for supporting the legal capacity of someone with a mental disability, in England and Wales. The framework maps out six models of undue influence and their salient features, when considered against the normative background of the CRPD. Doing so draws out key issues that policy makers must navigate in developing an approach to undue influence for the support context. These largely concern the inclusion of certain potential features as central: modes of influence, wrongful action by the supporter, a disadvantageous or harmful outcome for the decision-maker, the decision-maker’s mental or physical state at the time of the decision, the construct of mental incapacity, the construct of vulnerability, and the construct of discursive control. In addition, two broader issues concern whether a single approach to undue influence will be appropriate for all kinds of supported decision and the normative foundations of the approach to undue influence in support relationships. Each model, its essential features and arising normative considerations are outlined in turn.

*Modes of influence model:* The characterisation of undue influence endorsed by the Committee on the Rights of Persons with Disabilities is limited to certain modes of influence, many of which are wrongful and may harm the supported person. A focus on certain modes of influence would limit the scope of the undue influence remedy, thereby restricting this power to vitiate decisions. For those who are committed to the importance of giving supported decisions legal standing and effect, this consequence may be appealing. However, much less appealing will be the risk that supporter influence could be profound and invasive but not deemed undue because the influence did not involve obvious threat, manipulation, and so on. In relation to this consequence, the concern involves supporters playing *either* a problematic paternalistic *or* a problematic harmful role. This limitation of the modes-of-influence approach seems significant and is avoided by other models.

*Will-based model:* On the will-based approach, originating in property and contract law, undue influence is conceptualised in terms of the relationship between the expressed decision and the decision-maker’s will. Any mode of influence might be undue and undue influence need not involve wrongful conduct or a harmful outcome. These features of a will-based model seem advantageous for the support context, as a way of protecting the decision maker against the inappropriate involvement that does not involve a harm or wrongful conduct by the supporter. However, a straightforward application of the ‘overborne will’ standard would allow a degree of influence by supporters that may be deemed inappropriate given their role. This concern appears to be recognised in the MCA Code, where ‘undue pressure’ by someone in a support role is given a wider characterisation, which more easily allows pressure to be deemed undue. Nonetheless, considered in relation to the other identified models, this is one that seems potentially fitting for the support context.

Lord Donaldson’s guidance regarding when a person’s will is overborne highlights three areas to be given consideration: (i) the strength of the person’s will, including factors such as exhaustion, pain or depression; (ii) the relationship between the people in question, with influence often being more powerful in certain kinds of relationships such as parent and child; and (iii) that certain kinds of arguments can be particularly forceful, for example, those based on religious or cultural norms and expectations. As well as their potential relevance for an approach to undue influence in the context of support, these considerations may be useful as a basis for developing guidance on avoiding inappropriate influence within support relationships. On this approach, findings of undue influence do not rely, either directly or indirectly, on the influenced person having a disability. Nonetheless, concerns might be raised about factors such as depression playing disproportionate part in the vitiating of supported decisions in practice.

*Inference-based model*: The law in England and Wales allows, in many contexts, that undue influence need not be proven and can, instead, be inferred from the situation. The support relationship has the features that underpin this approach, indicating its appropriateness for this context: trust and confidence, and ascendency. The key question in relation to this model concerns what features of the situation would warrant an inference to undue influence by a supporter. What features would ‘call for an explanation’? A wrongful action or disadvantageous outcome is often implicated in existing law. However, linking undue influence in the support context to these features would be in tension with the shift away from paternalistic approaches to people with disabilities, on some interpretations of the CRPD. The supported person would not be protected against influence that is experienced as invasive but has a beneficial outcome, and their abilty to make risky decisions would be contrained. A normative question is therefore raised about the importance of avoiding these consequences. Nonetheless, this is another model that seems potentially fitting for the support context.

*Incapacity-based model:* Several cases have explored the idea of combining the overborne will standard with the construct of mental incapacity. On this approach, the influence of others is considered in terms of its impact on the influenced person’s ability to ‘weigh’ the decision. This model suggests new ways of thinking about undue influence in terms of psychological capacities, for example, regarding the importance of the ‘ability to conceive of separateness as viable’.^140^ However, the intimate link between the notion of mental incapacity and psychological impairment raises a question about whether this model could be implemented in a disability-neutral way. It also raises a more fundamental concern about what the notion of mental incapacity means for how we understand the problem of undue influence. It was argued that this approach inclines us to think about a fundamentally interpersonal problem, in terms of the influenced person’s impaired decision-making ability. This seems an undesirable consequence for anyone who is unduly influenced, including people with a mental disability.

*Will and vulnerability-based model:* Undue influence cases that have invoked the High Court’s inherent jurisdiction since the implementation of the MCA, have adopted the overborne will standard in connection with the concept of vulnerability. In this context, ‘vulnerability’ is often explained with reference to intrinsic features of the person, such as borderline mental capacity, which may raise concerns about whether, in practice, such a model would be disability neutral. Crucially, the overborne will standard operates in property, contract and medical law without reference to vulnerability, raising a question about what this construct adds to a will-based model. Due to the above concern about the potential for unjustified discrimination against people with disabilities, a compelling answer would seem to be required to justify applying this approach in the support context.

*Discursive control model*: Motivated by the characterisation of ‘undue pressure’ set out in the MCA Code of Practice, this novel approach to conceptualising undue influence could allow greater scope for vitiating a decision relative to the overborne will standard. It might, nonetheless, be implemented in a way that draws on Lord Donaldson’s guidance to highlight the circumstances that involve a particular risk of undue influence. On the discursive control approach, what makes supporter involvement unduly influencing is its impact on discourse about the relevant decision: either the person’s discourse within their own thinking or within the support relationship. This model keeps the interpersonal nature of undue influence in sight, and does not rely, directly or indirectly, on the notion of disability. It also generates practical recommendations for avoiding problematic influence. A key normative question concerns whether such an approach would too readily allow supported decisions to be vitiated. This is the final model identified as potentially fitting for the support context.

This mapping of salient features associated with the identified models indicates that three of the six models are a potential fit for conceptualising undue influence within the support relationship, when considered against the normative background of the CRPD. However, each comes with consequences to be accepted. The overborne will standard would allow significant scope for supporter influence before a decision could be vitiated; an inference-based model would make the harmfulness of supported decisions central to questions of undue influence; and a discursive control model may allow significant scope for denying the legal standing or effect of supported decisions.

The first of the two broader issues identified in this article concerns whether it would be appropriate to adopt a single approach to undue influence across all supported decisions. Similar concerns were expressed in medical cases. Nonetheless, a relatively uniform approach has been adopted in England and Wales across decisions concerning property, contracts and medical consent. This suggests that a single approach may be plausible across a range of supported decisions. However, courts have indicated that certain factors, such as a decision being life-threatening, may justify applying the standard more strictly. If this approach is adopted in the support context, key questions will concern how much outcome-dependent flexibility is desirable, and what kinds of outcomes justify this flexibility. For example, how important is the protection of life, wellbeing or finances, in the context of supported decision-making? This links to the second broader issue, which concerns the normative commitments underpinning the development of law in this area.

## XI. THE NORMATIVE FOUNDATIONS OF THE SUPPORT RELATIONSHIP

Developing an approach to undue influence for the support context ultimately involves a great many variables related to the broader political and legal context, which fall outside the scope of this article. The aim here has been to bring out the interplay between normative perspectives regarding the support relationship, and various possibilities for conceptualising undue influence in this context. This analysis has identified three models as potentially fitting for the support relationship. Further deliberation about the appropriateness of these models requires a deeper engagement with the normative implications associated with each. One crucial question for policy makers, therefore, concerns the values that should be fundamental to the provision of support, and what they mean for an approach to undue influence in this context.

Towards addressing this normative question, the analysis has set out the complexities that are involved. It was argued that the values found in the CRPD fail to provide clear answers to the relevant policy questions. For example, the requirement to promote the inherent dignity, freedom to make one’s own choices and independence of persons with disabilities can be used to justify either an approach to undue influence that more readily, or less readily, allows decisions to be vitiated. The further questions that are raised include: who is the greater threat to the dignity, freedom and independence of persons with mental disabilities, the supporter or the state; and whether, and to what extent, protection in the form of an undue influence remedy is an appropriate response to unjustified paternalism as well as harm?

What emerges is that the essential normative question is not a straightforward matter of finding the proper balance between freedom and protection for people with mental disabilities in the context of supported decision-making. The conversations and wide engagement with stakeholders that will enable the normative foundations of the support relationship to evolve, must be about more fine-grained questions that specify, for example, freedom from whom, and protection in relation to what.

## Footnotes

1 The CRPD requires that States Parties, ‘take appropriate measures to provide access by persons with disabilities to the support they may require in exercising their legal capacity’. (Art 12(3)). This is coupled with a requirement to ensure that, ‘persons with disabilities enjoy legal capacity on an equal basis with all others in all aspects of life’. (Art 12(2)). Legal capacity is understood to involve the person having legal standing, as a ‘holder of rights’, as well as being a legal agent, whose decisions have legal effect. See also: Committee on the Rights of Persons with Disabilities, *General Comment No 1, Article 12: Equal recognition before the law* (United Nations 2014) paras 3, 12. The development and interpretation of Article 12 has been extensively discussed: A Kanter, *The Development of Disability Rights Under International Law* (Routledge, 2017) 235–90; L Series and A Nilsson, ‘Article 12 CRPD: Equal Recognition Before the Law’ in I Bantekas, MA Stein and D Anastasiou (eds), *The UN Convention on the Rights of Persons with Disabiltiies: A Commentary* (OUP, 2018) 339–82.

2 In General Comment No 1, the Committee on the Rights of Persons with Disabilities adopted a strict interpretation of Article 12 according to which substituted decision-making based on mental impairment or mental incapacity is never permissible: ‘Article 12 … makes it clear that “unsoundedness of mind” and other discriminatory labels are not legitimate reasons for the denial of legal capacity … [and] perceived or actual deficits in mental capacity must not be used as justification for denying legal capacity.’ (n 1) para 13. Concerns in response to this position have been raised by many commentators including: J Dawson, ‘A Realistic Approach to Assessing Mental Health Laws’ Compliance with the UN Convention on the Rights of Persons with Disabilities’ (2015) 38 International Journal of Law and Psychiatry*;* M Burch ‘Autonomy, Respect and the Rights of Persons with Mental Disabilities in Crisis’ (2017) 34 Journal of Applied Philosophy 389; P Appelbaum, ‘Saving the UN Convention on the Rights of Persons with Disabilities – from itself’ (2019) 18 World Psychiatry 1. In a recent development, noted by Alex Ruck Keene, the Committee’s concluding observations on Australia (among other countries) in September 2019, indicate a shift in its position, given the endorsement of the Autralian Law Reform Commission’s proposals, which retain some substituted decision-making. <https://www.mentalcapacitylawandpolicy.org.uk/the-crpd-committee-and-legal-capacity-a-step-forwards/>, 14 October 2019; accessed 30 Jaunary 2020.

3 See the Scottish and Australian policy documents discussed below. For a review of relevant documents in England and Wales: A Alley and others (forthcoming) Guidance on support for decision-making in England and Wales: A pragmatic review.

4 For example, support may involve an interpretation of the person’s will and preference. M Bach and L Kerzner, *A New Paradigm for Protecting Autonomy and the Right to Legal Capacity* (Law Commission of Ontario 2010) 77–78.

5 In Victoria, Australia, those in ‘supportive guardian’ or supportive administrator’ roles can be ‘granted authority to get information from others about the person [they are supporting] to help the person make a decision. For example, from doctors, banks, utility providers, disability organisations or government agencies’. <https://www.publicadvocate.vic.gov.au/guardianship-administration/supportive-guardians-and-supportive-administrators> accessed 15 October 2020.

6 This is a crucial and vexing question for policy makers. Gooding and Flynn argue for the importance of allowing supported decisions to have adverse effects, on grounds of the individual’s ‘dignity of risk’. P Gooding and E Flynn, ‘Querying the Call to Introduce Mental Capacity Testing to Mental Health Law: Does the Doctrine of Necessity Provide an Alternative?’ (2015) 4 Laws 245, 261–62. Bach and Kerzner adopt a more cautious approach. Bach and Kerzner (n 4) 47.

7 One exception is the Canadian Provence of Alberta’s Adult Guardianship and Trustees Act (2008), which provides that, ‘a person may refuse to recognize a decision made or communicated by or with the assistance of a supporter as the decision of the supported adult if the person has reasonable grounds to believe that (a) the supporter exercised undue influence on the supported adult to make the decision’ (s 6(2)). To my knowledge, the meaning of undue influence in this context has not yet been explored in case law. For an overview of law concerning undue influence in Canadian jurisdictions, see: M Bach and L Kerzner, *Supported Decision-Making: A Roadmap for Reform in Newfoundland & Labrador* (Institute for Research and Development on Inclusion and Society, 2020) 93–95.

8 L Series, ‘Relationships, Autonomy and Legal Capacity’ (2015) 40 *International Journal of Law and Psychiatry* 81, 88–89; W Martin and others, *Three Jurisdictions Report* (Essex Autonomy Project, 2016) 84–86; T Carney, ‘Prioritising Supported Decision-making: Running on Empty or a Basis for Glacial-to-Steady Progress?’ (2017) 6 *Laws*, doi:10.3390/laws6040018, 10; Series and Nilsson (n 1) 370–71.

9 General Comment No 1 elaborates on the potential scope of ‘support’, which is said to include measures relating to communication, information provision, advance planning and advocacy (n 1) s 17. Penzenstadler and colleagues’ review of support models in mental healthcare practice include support in the decision-making process, advance directives and peer advocacy. L Penzenstadler, A Modolynski and Y Khazaal, ‘Supported Decision-making for People with Mental Health Disorders in Clinical Practice: A Systematic Review’ (2019) *International Journal of Psychiatry in Clinical Practice*  https://doi.org/10.1080/13651501.2019.1676452.

10 R Morgan and J Devenney, ‘Mrs U v Centre for Reproductive Medicine [2002] EWCA Civ 565’ (2003) 25 *Journal of Social Welfare and Family Law* 74; see also: C Stewart and A Lynch, ‘Undue Influence, Consent and Medical Treatment’ (2003) 96 *Medicine and Law* 598.

11 J Craigie, ‘A Fine Balance: Reconsidering Patient Autonomy in Light of the UN Convention on the Rights of Persons with Disabilities’ (2015) 29 *Bioethics* 398.

12 Here I present the evaluative landscape as involving two positions. In forthcoming work, Paul Skowron and Matt Matravers present a more complex picture.

13 As discussed below. People First (Scotland), *Supported Decision Making: A Framework* (People First (Scotland), 2017).

14 M Verkerk, ‘A Care Perspective on Coercion and Autonomy’ (1999) 13 *Bioethics* 358. Through empirical work, Knox and colleagues identified that understanding what the supported person values is a key element of the support relationship, and they note that ‘it was … clear that self-conceptualisation is shaped by feedback from others’: L Knox, JM Douglas and C Bigby, ‘Becoming a Decision-making Supporter for Someone with Acquired Cognitive Disability Following Traumatic Brain Injury’ (2016) 3 *Research and Practice in Intellectual and Developmental Disabilities* 12, 16.

15 The meaning of ‘will and preferences’ in this context has attracted considerable academic discussion: G Szmukler, ‘The UN Convention on the Rights of Persons with Disabilities: “Rights, will and preferences” in Relation to Mental Health Disabilities’ (2017) 54 *International Journal of Law and Psychiatry* 90; Series and Nilsson (n 1) 369–70; P Skowron, ‘Giving Substance to “the best interpretation of will and preferences”’ (2019) 62 *International Journal of Law and Psychiatry* 125; T Carney and others ‘Realising “Will, Preferences and Rights”: Reconciling Differences on Best Practice Support for Decision-Making?’ (2019) *Griffith Law Review*, DOI: https://doi.org/10.1080/10383441.2019.1690741.

16 For example: P Bartlett and M Schulze, ‘Urgently Awaiting Implementation: The Right to be Free from Exploitation, Violence and Abuse in Article 16 of the Convention on the Rights of Persons with Disabilities (CRPD)’ (2017) 53 *International Journal of Law and Psychiatry* 2. This article is part of a special issue dedicated to Article 16.

17 Rosemary Kayess and Robert French describe the idea of the ‘paradigm shift’ embodied in the CRPD as a move ‘away from a social welfare response to disability to a rights-based approach’, involving a ‘shift in attitudes’ to see disabled people not as objects in need of protection or treatment, but rather as equal members of society with rights: R Kayess and R French, ‘Out of the Darkness into the Light? Introducing the Convention on the Rights of Persons with Disabilities’ (2008) 8 *Human Rights Law Review* 1, 3.

18 C Kong, *Mental Capacity in Relationship: Decision-Making, Dialogue and Autonomy* (CUP, 2018).

19 Martin and others (n 8) 45.

20 Art 1.

21 E Flynn and A Arstein-Kerslake, ‘Legislating personhood: Realising the Right to Support in Exercising Legal Capacity’ (2014) 10 *International Journal of Law in Context* 81, 82 and 84.

22 P Jaarsma and S Welin, ‘Autism as a Natural Human Variation: Reflections on the Claims of the Neurodiversity Movement’ (2012) 20 *Health Care Analysis* 20.

23 J Craigie and L Bortolotti, ‘Rationality, Diagnosis and Patient Autonomy in Psychiatry’ in JZ Sadler, KWM Fulford and W van Staden (eds), *The Oxford Handbook of Psychiatric Ethics* (OUP, 2015).

24 Indeed, this is what Chen-Wishart observes in her analysis of Singaporean undue influence cases. M Chen-Wishart, ‘Legal Transplant and Undue Influence: Lost in Translation or a Working Misunderstanding?’ (2013) 62 *International and Comparative Law Quarterly* 1, 28.

25 Chen-Wishart presents her account of the purpose of undue influence law in the following way: ‘To uphold the integrity of such valuable relationships, the law requires the defendant to have due regard for the *substantive and procedural norms implicit in the relationship of influence* he shares with the claimant when he transacts with her.’ M Chen-Wishart, ‘Undue Influence: Beyond Impaired Consent and Wrongdoing Towards a Relational Analysis’ in *Mapping the Law: Essays in Memory of Peter Birks* (OUP, 2006) 201, 203; author’s emphasis.

26 Office of the Public Advocate, *Supported Decision Making: background and discussion paper* (Office of the Public Advocate, 2009) s 8.8.

27 (n 1) para 22.

28 ibid, para 22.

29 Here ‘wrongdoing’ is used to indicate that the action involves a wrong, independent of the consequences, such that strong reasons will be required to make the action morally justified. This description arguably applies to threat, deception and manipulation; and could also apply to aggression and the generation of fear, in the decision-making support context.

30 Harm seems a likely consequence of these modes of influence in many situations, but it is not necessary for undue influence on the General Comment No 1 account. For example, a person may be deceived through the withholding of information, to avoid a harm. It can be argued that they are wronged, despite not being harmed, as explored in the film ‘The Farewell’ (2019) in which a grandmother is not told about her terminal cancer diagnosis.

31 The review involved web-based searches and consultation with experts and practitioners involved in supported decision-making research and policy development in the UK, Canada, and Australia.

32 Mental Welfare Commission Scotland and Centre for Mental Health and Mental Capacity Law, *Scotland’s Mental Health and Capacity Law: A Case for Reform* (Mental Welfare Commission Scotland, 2017) para 6 (a) (4); authors’ emphasis.

33 ibid, para 6(a)(4).

34 Mental Welfare Commission for Scotland and J Stavert, *Supported Decision Making* (Mental Welfare Commission for Scotland, 2016).

35 ibid, s 5.2.3.

36 L Duffield and others, *Dealing with Challenges in Providing Decision-making Support: Information Sheet for Direct Support Workers* (Scope 2016) 1.

37 Noting the controversy surrounding the interpretation of this phrase, discussed at n 15. Committee on the Rights of Persons with Disabilities (n 1) para 17.

38 People First (Scotland), *Supported Decision Making: A Framework* (People First (Scotland), 2017).

39 ibid 24.

40 Ibid 25.

41 P Birks, ‘Undue Influence as Wrongful Exploitation’ (2004) 120 *Law Quarterly Review* 134, 136; see also: P Birks and NY Chin, ‘On the Nature of Undue Influence’ in J Beatson and D Friedman (eds), *Good Faith and Fault in Contract Law* (OUP 1995).

42 *Royal Bank of Scotland PLC v Etridge (No 2)* [2002] 2 AC 773, [6].

43 ibid para 6.

44 ibid para 7.

45 Scottish law concerning undue influence diverges somewhat from English and Welsh law. Martin and others (n 8) 44–48.

46 *Hall v Hall* (1868) LR 1 P & D 481, 482.

47 Edwards (Deceased), Re [2007] EWHC 1119 (Ch).

48 Terry Carney has expressed the concern that will-based common law principles, ‘do not readily lend themselves to simple application outside the higher courts’. (n 8) 10.

49 Bach and Kerzner draw a parallel between the undue influence jurisprudence in Canadian jurisdictions, which is based on the person’s ‘true intentions’ and ‘free will or volition’, and the emphasis on respect for the person’s ‘will and preferences’ in General Comment No 1 from the UN Committee on the Rights of Persons with Disabilities (n 7) 25.

50 *London Borough of Brent and NB, SA, AD, MB, SB* [2017] EWCOP 34. This case raises a question about whether the Court would have been willing to adopt this approach, had the decision concerned a life-threatening treatment refusal. Subsequent hearings concerning MB’s healthcare continued to place significant weight on his expressed wishes and feelings in the determination of his best interests, despite the concerns about his mother’s influence. However, MB did not object to the procedures recommended by medical professionals, and these were found to be in his best interests. *Imperial College Healthcare An NHS Trust and MB and Others* [2019] EWCOP 29, [14], [25].

51 *London Borough of Brent and NB*, ibid. The Court concluded that due to the enmeshed relationship, ‘MB is unable or unwilling to express views that differ from his mother’s’ ([169f], but that MB ‘does indeed hold those strong wishes and feelings’ [173] and these were therefore said to be decisive in relation to his best interests. This position suggests that in terms of the legal standing of MB’s wishes, it was not the degree to which these were influenced that mattered but whether he experienced the expressed wishes as his own.

52 *Etridge* (n 42) [9]; *Allcard v Skinner* (1887) 36 Ch.D. 145.

53 *Etridge*, ibid, [8]–[25].

54 Ibid, [11], [157]. Chen-Wishart cites several cases involving ‘dependency because of physical or mental deterioration’ or dependency and trust ‘placed in an employee carer’: M Chen-Wishart, ‘Undue Influence: Vindicating Relationships of Influence’ (2007) 59 *Current Legal Problems* 231, 234 and 237.

55 This claim is compatible with the idea that some degree of vulnerability is an intrinsic part of being human; it does not imply a necessary link between having a mental disability and being particularly vulnerable; and it allows that the vulnerability associated with mental disability may be due, at least in part, to social context. For overview: J Herring, *Vulnerable Adults and the Law* (OUP, 2016) 1–43; C Mackenzie, W Rogers and S Dodds, *Vulnerability* (OUP, 2014).

56 *Etridge* (n 42) [9].

57 The ‘presumption of influence’ can, alternatively, be established on a case by case basis: ibid [10].

58 Chen-Wishart (n 54).

59 *Etridge* (n 42) [156].

60 Ibid, [12], [24], [25], [156].

61 Birks (n 41) 35; Chen-Wishart (n 25) 211.

62 Chen-Wishart (n 54) 265.

63 Birks (n 41) 136; Birks and Chin (n 41).

64 Chen-Wishart (n 25) 206.

65 Chen-Wishart (n 24) 9. *Allcard v Skinner* (n 52) is often cited in which the Mother Superior of a convent was found to have unduly influenced a nun’s decision make a considerable gift to the convent, despite the Mother Superior being found to have done no wrong.

66 *Re T (Adult refusal of treatment)* [1993] Fam 95.

67 The refusal of blood products was life-threatening, but there was no suggestion that the mother’s pressure was malicious.

68 As beautifully explored in the play ‘Jellyfish’ by Ben Weatherill. <https://www.nationaltheatre.org.uk/shows/jellyfish> accessed 15 October 2020.

69 If support structures are set up such that a significantly harmful or unfair outcome always ‘calls for an explanation’, then a presumption of undue influence would arise in the context of many apparently risky or unwise supported decisions, which may also raise concerns from a human rights perspective. P Gooding, ‘Supported Decision-Making: A Rights-Based Disability Concept and its Implications for Mental Health Law’ (2013) 20 *Psychiatry, Psychology and Law* 431, 435–36.

70 United Nations (n 1) Art 12(4). The standard way to rebut inferred undue influence is to show that the decision maker had independent legal advice, although the court must be satisfied that the advice provided, ‘the necessary independence of judgement and freedom to make choices with a full appreciation of what he was doing’. *Pesticcio v Huet* [2004] EWCA Civ 372. This discussion suggests that independent advice from a third party may be particularly important when the person’s decision involves a risk of significant harm. This kind of oversight could be provided by a second supporter. Alternatively, it might be argued that that this advice should involve a professional with relevant medical, financial or legal expertise.

71 Morgan and Devenney (n 10) 76.

72 *Re T* (n 66) 119, 121.

73 *Mrs U v Centre for Reproductive Medicine* [2002] EWCA Civ 565, [25].

74 *Edwards v Edwards* [2007] EWHC 138; *Craig v Lamoureux* [1920] AC 349, 357.

75 Stewart and Lynch (n 10); Morgan and Devenney (n 10).

76 *Re T* (n 66). The term ‘capacity’ here is used to indicate that this area of law includes broader grounds for a loss of legal capacity beyond mental capacity.

77 ibid 107–08.

78 ibid 111.

79 ibid 111–12. The statement that, ‘the influence of her mother was such as to vitiate the decision which she [T] expressed’, indicates that this was a case of actual rather than presumed undue influence.

80 ibid 113–14.

81 As pointed out in *DL v A Local Authority & Others* [2012] EWCA Civ 253 (*Re L*), [57]–[58].

82 *Mrs U* (n 73).

83 ibid, [28], judge’s emphasis. The Court cited Lord Donaldson’s guidance, that ‘it does not matter how strong the persuasion was *so long as it did not overbear the independence of the patient's decision’ Mrs U* (n 73) [28], judge’s emphasis.

84 In place of the ‘overborne will’ standard it was proposed that the question should be ‘whether [Mr U] had a real choice on that day to refuse to alter the form’: ibid, [22].

85 ibid, paras [24], [26].

86 ibid, [20]. The basis for the agreement was not explained, but it seems likely that a ‘presumption of influence’ did not arise in the context of the relationship between Mr U and the nurse who persuaded him to withdraw his consent.

87 ibid, [26].

88 ibid, [25].

89 *Southend-on-Sea Borough Council and Mr Meyers* [2019] EWHC 399 (Fam), [42]. This case concerned the impact of an ‘entirely dysfunctional relationship’ (para 34) between an elderly father and his son, and the impact of the son’s ‘insidious and pervasive’ ([41]) influence on the father’s decisions about living together, as discussed below.

90 *A Local Authority and Mrs A and Mr A* [2010] EWHC 1549 (Fam).

91 Since October 2007, all questions of mental capacity have been governed by the MCA.

92 *Re A* (n 90) [6].

93 MCA s 3(1).

94 MCA s 3(2).

95 *Re A* (n 90) [66], my emphasis.

96 ibid, [73].

97 ibid, [73].

98 ibid, [73]. Factors including: ‘Mrs A’s dependence on him and fear of rejection; her suggestibility and wish to please him; his lack of insight as to the true extent of her difficulties; his rigid views about their status as husband and wife; his own wish to start a family, which is to be fully respected; and the fact that he has never yet felt included the decision.’

99 ibid, [66].

100 For example, one expert proposed that the ‘ability to conceive of separateness as viable’ is a component of the ability to ‘weigh’ information within the MCA’s functional test. *London Borough of Brent and NB* (n 50) [71].

101 ‘Under article 12 of the Convention, perceived or actual deficits in mental capacity must not be used as justification for denying legal capacity.’ Committee on the Rights of Persons with Disabilities (n 1) para 13.

102 W Martin and others, ‘Achieving CRPD-Compliance: Is the Mental Capacity Act of England and Wales Compatible with the UN Convention on the Rights of Persons with Disabilities? If not, what next?’ (2014) <http://repository.essex.ac.uk/13624/1/EAP-Position-Paper-FINAL-copy.pdf> accessed 15 October 2020, 31–36.

103 Relatedly, see Chen-Wishart’s critique of claimant-sided views of undue influence, as demeaning the influenced party (n 25) 206.

104 Series (n 8) 84.

105 *London Borough of Brent and NB* (n 50). Another example is *The London Borough of Redbridge v G, C &* F [2014] EWHC 485 (COP). This case concerned an elderly woman’s capacity to make decisions in areas including contact and cohabitation with two people acting as her carers, and their influence over her. The concerns about influence were based on reports about G’s live-in carers’ use of controlling behaviour, threats and physical abuse. The Court found that G lacked mental capacity in several areas due to a significant cognitive impairment consistent with dementia, in accordance with the MCA (paras 54, 77). The impairment was said to compromise her ability to weigh information in decisions about finances, contact and living arrangements with her carers, with these difficulties made ‘harder for her because of the influence of [the carers]’ ([55], [81]). The Court rejected the idea that G lacked capacity solely due to this influence, but accepted that, ‘the dependency and the influence inhibit G's ability to reach decisions and undermines her capacity to do so’ ([60], [79]).

106 *London Borough of Brent and NB* (n 50) [172].

107 ibid, [70].

108 ibid, [71]; The expert also suggested that addressing this issue through therapy might mean that MB ‘may weigh up slightly better’ ([73]).

109 ibid, [51].

110 *London Borough of Brent and NB* (n 50) [169].

111 *Re L* (n 81) [64]. ‘In the context of the inherent jurisdiction I would treat as a vulnerable adult someone who, whether or not mentally incapacitated, and whether or not suffering from any mental illness or mental disorder, is or may be unable to take care of him or herself, or unable to protect him or herself against significant harm or exploitation, or who is deaf, blind or dumb, or who is substantially handicapped by illness, injury or congenital deformity. This, I emphasise, is not and is not intended to be a definition. It is descriptive, not definitive; indicative rather than prescriptive’: *A local Authority and MA, NA, SA* [2005] EWHC 2942 (Fam) (*Re SA (Vulnerable Adult with Capacity: Marriage)*, [77], [82].

112 *Re L* (n 81) [1].

113 ibid, [20], [65].

114 ibid.

115 *Southend-on-Sea Borough Council and Mr Meyers* (n 89).

116 *A local Authority v DL* [2011] EWHC 1022 (Fam), [23].

117 The Court held that the parents ‘plainly had mental capacity to make decisions for themselves’ including decisions regarding their son. *Re L* (n 81) [3].

118 *A Local Authority v DL* (n 116) para 6; *A local Authority v DL* [2010] EWHC 2675 (Fam), [4].

119 *Re SA* (n 111) [78]; *Re L* (n 81) [22].

120 *Re SA* concerned arrangements for a young woman’s marriage. SA was described as ‘profoundly deaf’ but functioning intellectually above ‘learning disability range’, the main difficulty being communication given her family’s inability to use British Sign Language (n 111) [8], [16].

121 *Re T* (n 66).

122 *Re SA* (n 111) [78]; discussed at *Re L* (n 81) [22].

123 *Re SA*, ibid, para 78; discussed in *Re L*, ibid, para 22.

124 *Southend-on-Sea Borough Council and Mr Meyers* (n 89).

125 ibid, [40].

126 ibid, [41].

127 ibid.

128 ‘The inherent jurisdiction is not confined to those who are vulnerable adults, however that expression is understood, nor is a vulnerable adult amenable as such to the jurisdiction. … [Rather,] it is likely to be easier to persuade the court that there is a case calling for investigation where the adult is apparently vulnerable than where the adult is not on the face of it vulnerable.’ *Re SA* (n 111) [83].

129 Series (n 8) 89.

130 Carney (n 8) 10.

131 *Southend-on-Sea Borough Council and Mr Meyers* (n 89)*;* See also *Re SA* (n 111) 164.

132 M Dunn, I Clare and A Holland, ‘To Empower or Protect? Constructing the “vulnerable adult” in English Law and Public Policy’ (2008) 28 *Legal Studies* 244. Beverly Clough acknowledges this and other concerns about the ‘vulnerable adult’ construct from a disability perspective, but contends that its use in social policy does not necessary have these consequences. B Clough, ‘Disability and Vulnerability: Challenging the capacity/incapacity binary’ (2017) 16 *Social Policy and Society* 469, 473–75. More strongly, Herring claims that use of the ‘vulnerable adult jurisdiction’ aligns closely ‘with the ideals in the [CRPD]’ because it minimises discrimination on grounds of mental disorder (Herring (n 55) 96).

133 MCA s 1(3).

134 For an analysis: Series (n 8).

135 Committee on the Rights of Persons with Disabilities, (n 1) para 13.

136 Department of Constitutional Affairs, *Mental Capacity Act 2005 Code of Practice* (The Stationary Office, 2007) s 2.8.

137 ibid s 2.8. The Code of Practice is currently being revised.

138 P Pettit, *A Theory of Freedom* (Polity Press, 2001) Ch 4; Craigie (n 11) 403–05.

139 Craigie (n 11) 405.

140 *London Borough of Brent and NB* (n 50) [71].

